# Identification of Key Genes and Pathways in Pancreatic Cancer Gene Expression Profile by Integrative Analysis

**DOI:** 10.3390/genes10080612

**Published:** 2019-08-13

**Authors:** Wenzong Lu, Ning Li, Fuyuan Liao

**Affiliations:** Department of Biomedical Engineering, College of Electronic and Information Engineering, Xi’an Technological University, Xi’an 710021, China

**Keywords:** pancreatic cancer, gene expression, hub gene, bioinformatics

## Abstract

Background: Pancreatic cancer is one of the malignant tumors that threaten human health. Methods: The gene expression profiles of GSE15471, GSE19650, GSE32676 and GSE71989 were downloaded from the gene expression omnibus database including pancreatic cancer and normal samples. The differentially expressed genes between the two types of samples were identified with the Limma package using R language. The gene ontology functional and pathway enrichment analyses of differentially-expressed genes were performed by the DAVID software followed by the construction of a protein–protein interaction network. Hub gene identification was performed by the plug-in cytoHubba in cytoscape software, and the reliability and survival analysis of hub genes was carried out in The Cancer Genome Atlas gene expression data. Results: The 138 differentially expressed genes were significantly enriched in biological processes including cell migration, cell adhesion and several pathways, mainly associated with extracellular matrix-receptor interaction and focal adhesion pathway in pancreatic cancer. The top hub genes, namely *thrombospondin 1*, *DNA topoisomerase II alpha*, *syndecan 1*, *maternal embryonic leucine zipper kinase* and *proto-oncogene receptor tyrosine kinase Met* were identified from the protein–protein interaction network. The expression levels of hub genes were consistent with data obtained in The Cancer Genome Atlas. *DNA topoisomerase II alpha*, *syndecan 1*, *maternal embryonic leucine zipper kinase* and *proto-oncogene receptor tyrosine kinase Met* were significantly linked with poor survival in pancreatic adenocarcinoma. Conclusions: These hub genes may be used as potential targets for pancreatic cancer diagnosis and treatment.

## 1. Introduction

In modern medicine, pancreatic cancer is one of the most difficult diseases to diagnose because of the early development of systemic metastatic disease. Although the incidence of pancreatic cancer is increasing, awareness of pancreatic cancer is relatively low. The 5-year survival rate of pancreatic carcinoma was about 8%, much less than that of other cancers [1]. The perspective of pancreatic cancer patients has not elevated notably although surgical methods and pharmaceuticals have enhanced treatment to some extent. Moreover, lower enrollment on clinical trials has resulted in a decrease of new therapy development [2]. Generally, some factors that may increase the risk of pancreatic carcinoma including pancreatitis, family history of pancreatic cancer, obesity, and older age so on. One of the main challenges of pancreatic carcinoma chemotherapy is the development of new therapeutic ways affording the elimination of tumors cells while sparing normal tissues. It may ameliorate the depressing outcome of pancreatic carcinoma by molecularly targeted therapeutic approaches used for aberrant signaling pathway in pancreatic cancer cells. Consequently, a relevant molecular target needs to be identified.

Microarray is one of the most recent advances being used for cancer research. Tumor formation involves aberrant changes in numerous cells and variations in genes. Microarray can help peculiarly in the identification of target genes of tumor suppressors and cancer biomarkers, and classification of tumors [3]. In recent investigations, numerous differentially expressed genes (DEG) have been identified through microarray in pancreatic carcinoma, and several potentially pivotal biomarkers were disclosed [4,5,6]. For instance, some key biomarkers have been exposed in pancreatic carcinoma, namely *intercellular adhesion molecule 2* (*ICAM2*), *anoctamin 9* (*ANO9*), *proline-rich tyrosine kinase 2* (*PYK2*) and *cyclin-dependent kinase 9* (*CDK9*) [7,8,9,10]. However, a different biomarker was uncovered in different research lab. Accordingly, there was no responsible biomarker in gene expression profile research of pancreatic carcinoma. The integrative bioinformatics method connecting with gene expression profiling technology might solve the deficiencies.

In this study, we used the publicly available microarray data sets of human pancreatic tissue and performed integrative analysis on DEG by bioinformatics analysis. Our results will disclose the particular biomarker and the underlying therapeutic target for pancreatic carcinoma.

## 2. Materials and Methods

### 2.1. Microarray Data

Four publicly available gene expression profiles (GSE15471, GSE19650, GSE32676 and GSE71989) were downloaded from the Gene Expression Omnibus (GEO) database and used in this study. Criteria of the selected dataset was as follows: (1) the GEO platform (GPL) is GPL570 (Affymetrix Human Genome U133 Plus_2.0 Array); (2) the number of samples is more than 20 containing normal and cancer tissues; (3) the samples are human pancreatic cancer tissue. The dataset of GSE15471 contained pancreatic tissue samples of 39 cancer patients and 39 healthy subjects. The dataset of GSE19650 contained pancreatic tissue samples of 15 cancer patients and 7 healthy subjects. The dataset of GSE32676 contained pancreatic tissue samples of 25 cancer patients and 7 healthy subjects. The dataset of GSE71989 contained pancreatic tissue samples of 13 cancer patients and 8 healthy subjects. Data of chronic pancreatitis tissue samples in GSE71989 were not included in this study. These four datasets were chosen for integrative analysis in this study including 92 pancreatic cancer samples and 61 healthy subjects.

### 2.2. Data Preprocessing and DEG Screening

Affy package of R language was used for manipulating the raw data following a 3-step process: background-adjusted, normalized, and log-transformed the raw data values [11]. Afterwards, the expression matrix with gene level was gained by transforming the expression matrix with probe level grounded on annotation files. DEG analysis was performed with multiple linear regression Limma package [12]. It estimates the fold changes and standard errors by fitting a linear model for each gene by lmFit and the empirical Bayes statistics implemented by eBayes, topTable etc. Statistical significance was set at *p* value <0.01 and log2-fold change (log2|FC|) > 1 for each dataset. In the following study, intersection of the 4-dataset DEG was defined as common DEG. A Venn diagram was used for showing the common DEG by VennDiagram package of R language. We further analyzed the DEG of intraductal papillary-mucinous adenoma (IPMA), intraductal papillary-mucinous carcinoma (IPMC) and intraductal papillary-mucinous neoplasm (IPMN) for the GSE19650 dataset by the same method.

### 2.3. Hierarchical Clustering Analysis

Gene expression values were extracted from the expression profile for each dataset. A bidirectional hierarchical clustering heatmap was constructed using gplots package of R language for DEG in every dataset. Besides, the hierarchical clustering was performed by limiting the analysis only to the 138 common DEG obtained from the 4 datasets. We used heatmap.2 function in gplots package of R to draw the heat map. In heatmap.2, the expression value of gene is in the row and the sample is in the column. After normalizing the value of row, clustering settings are specified via distfun (method = ‘euclidean’) and hclustfun (method = ‘complete’) function.

### 2.4. Functional and Pathway Enrichment Analysis

On the basis of the database for Annotation, Visualization and Integrated Discovery (DAVID), common DEG were classified according to genes biological processes, molecular functions, or cellular components by gene ontology (GO) consortium reference [13]. The DAVID database was also used for performing pathway enrichment analysis with reference from kyoto encyclopedia of genes and genomes (KEGG) database. A cut-off point was delimited as *p* value < 0.05 and Benjamini-Hochberg false discovery rate (FDR) < 0.05. Moreover, we used KOBAS 3.0 (http://kobas.cbi.pku.edu.cn/anno_iden.php; Peking University: Beijing, China) to further perform GO and KEGG analysis.

### 2.5. Protein–Protein Interaction Network Construction and Hub Gene Analysis

The Search Tool for the Retrieval of Interacting Genes (STRING) version 10.5 (http://www.string-db.org/) was used for constructing the protein–protein interaction (PPI) networks [14]. The PPI network was constructed and visualized using cytoscape software version 3.5.0 (California, USA) for the common DEG [15]. The plug-in cytoHubba was used for exploring key nodes and fragile motifs in the PPI network by some topological algorithms including Degree, Edge Percolated Component, Maximum Neighborhood Component, Density of Maximum Neighborhood Component, Clustering Coefficient, Maximal Clique Centrality, Bottleneck, EcCentricity, Closeness, Radiality, Stress, and Betweenness [16]. A definition of 12 topological algorithms is described in the Supplementary Table S1. The top 30 nodes were considered as notable genes in the network for every topological analysis method. The intersected genes of top 30 nodes of every topological algorithm were regarded as the most important hub genes in the network.

### 2.6. Validation and Survival Analysis of the Hub Genes in The Cancer Genome Atlas (TCGA) Dataset

UALCAN is an interactive web-portal to perform to in-depth analyses of The Cancer Genome Atlas (TCGA) gene expression data (http://ualcan.path.uab.edu/index.html) and it uses TCGA level 3 RNA-seq and clinical data from 31 cancer types [17]. The correlation between hub genes expression and survival in pancreatic adenocarcinoma was analyzed by UALCAN. The patient objects with pancreatic adenocarcinoma were split into two groups according to the expression of a particular gene (high vs. low/medium expression). 

## 3. Results

### 3.1. Identification of DEG

**Figure 1:**
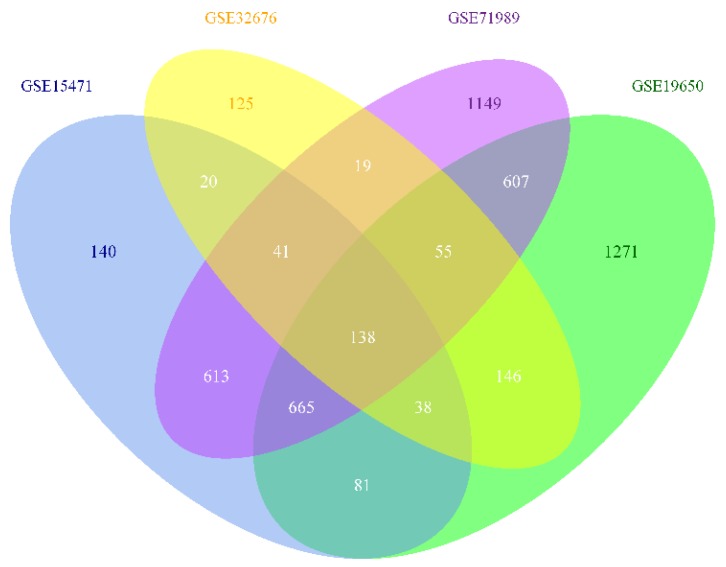
Four-set Venn diagram showing the common differentially expressed genes from the four Gene Expression Omnibus series datasets. Differentially expressed genes (DEG) were identified with classical t test, statistically significant DEG were defined with *p* < 0.01 and log_2_-fold change (log_2_FC) >1 or < −1 as the cut-off criterion for every dataset.

**Figure 2:**
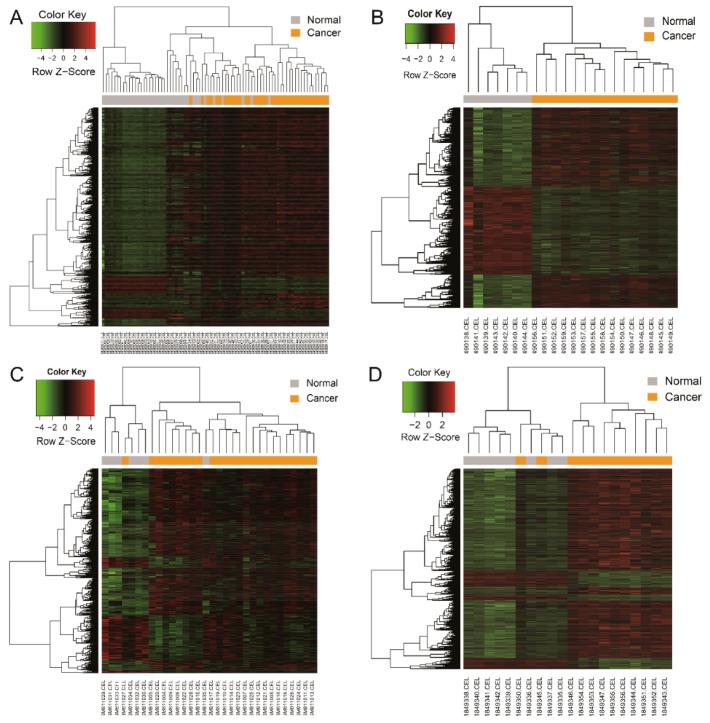
Heat map showing up-regulated and down-regulated differentially expressed genes in pancreatic adenocarcinoma compared to the normal samples in the four datasets. The expression values are log2 transformed for absolute value of fold changes (>1 or <−1) between normal tissues and pancreatic adenocarcinoma samples. Green represents down-regulation and red represents up-regulation. (**A**) GSE15471; (**B**) GSE19650; (**C**) GSE32676; (**D**) GSE71989.

A total of 138 common DEG were identified from the intersected parts of the four profile datasets including 93 up-regulated genes and 45 down-regulated genes in the pancreatic carcinoma samples compared to normal samples, which was exhibited by a Venn diagram (Figure 1). The gene expression value was extracted from every profile dataset and a hierarchical clustering heat map was plotted to show the DEG (Figure 2). In Figure 2, it can be seen that some cancer GSE samples (GSM) of the GSE15471 dataset were not classified as the cancer group. Similarly, this phenomenon also presents in the datasets of GSE32676 and GSE71989. Additionally, it shows that a clearer separation between cancer and normal samples (Supplementary Figure S1), which partly support the idea that these 138 genes can act as a pancreatic cancer signature.

The number of DEG of IPMA, IPMC and IPMN is 4021, 4047 and 4331, respectively. During the process, 47 IPMN specific genes were obtained by removing DEG of IPMA and IPMC from the 1271 genes. They are: *RPS4Y1/SCG5/NFIA/FABP6/RPL31/HHIP/KIF5C/LOC158402/BEX4/PID1/P2RY12/ GDAP1/ABI2/PRDM10/ATP7B/ZNF107/NAPRT/RPL11/FGA/ABCC4/ZDHHC9/SLC5A1/XIAP/SCGB1D2/LOC101928000/4-Sep/BTBD10/CHL1/XKR4/B3GALT2/ALG6/NBR2/RAD51AS1/ACOT8/DPP8/ ADSS/AGPS/ARL6IP1/CST3/TIPRL/MUM1/MSX2/C1orf53/BTBD3/H3F3A/NDUFB4/MPC2*.

### 3.2. GO Functional and Pathway Enrichment Analysis

As shown in Table 1, the DEG was significantly enriched in biological processes containing cell migration, cell adhesion, cell-cell adhesion, extracellular matrix disassembly and hemidesmosome assembly. GO analysis exhibited that the DEG was obviously enriched in extracellular exosome, plasma membrane, bicellular tight junction, focal adhesion for cell component. In addition, GO analysis also displayed that the DEG was markedly enriched in laminin binding, protein homodimerization activity, protein phosphatase binding and cadherin binding involved in cell-cell adhesion for molecular function. However, there are considerable differences in the ranked GO term by p-value between DAVID and KOBAS. For example, the term of cell migration is ranked first in the results of DAVID but it ranked 60 in the KOBAS analysis (Supplementary Table S2). 

The results of KEGG signaling pathway analysis showed that the DEG was markedly enriched in extracellular matrix (ECM)-receptor interaction, proteoglycans in cancer, focal adhesion, mucin type o-glycan biosynthesis and phosphoinositide 3-kinase- protein kinase B (PI3K- Akt) signaling pathway (Table 1). The results of KEGG pathway analysis are consistent with the KOBAS analysis.

### 3.3. PPI Network Construction and Hub Genes Identification

**Figure 3:**
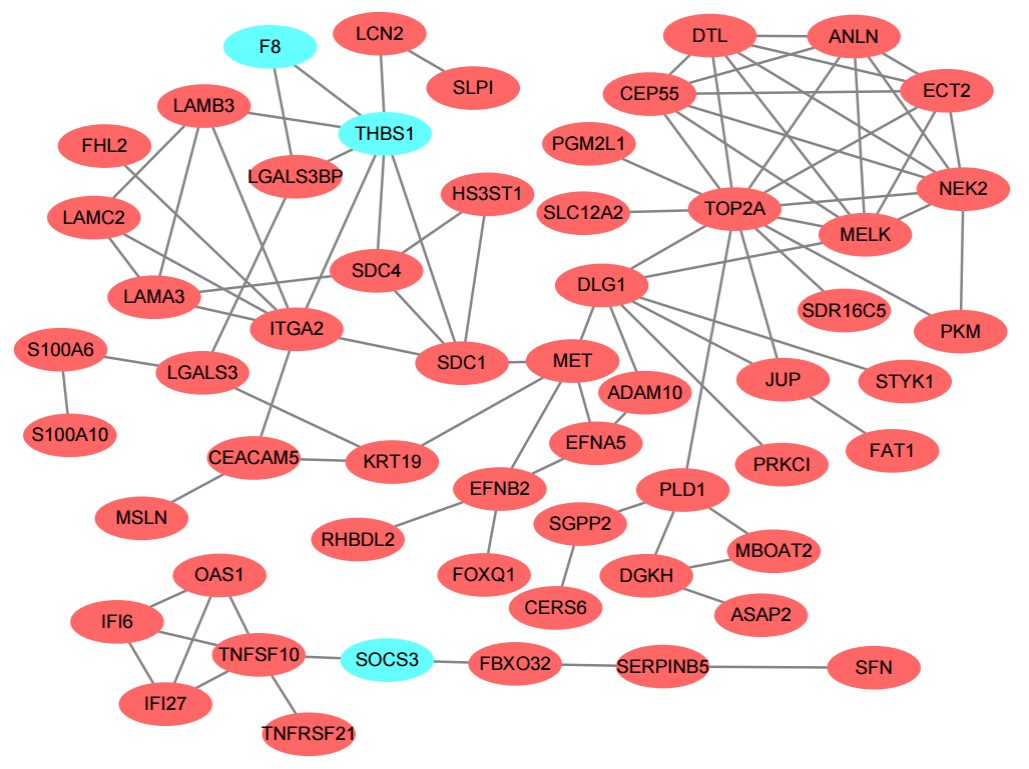
Differentially expressed genes protein–protein interaction (PPI) network was constructed and visualized using Cytoscape software. Red nodes represent up-regulated genes and baby blue nodes represent down-regulated genes in pancreatic adenocarcinoma compared to the normal samples. Only two nodes included one edge and alone node were removed from the network.

The PPI relationship was displayed in Figure 3. It was apparent from the figure that very few down-regulated genes are there. These genes were *thrombospondin 1* (*THBS1*), *coagulation factor VIII* (*F8*) and *suppressor of cytokine signaling 3* (*SOCS3*). In order to identify the key genes in the PPI relationship, 12 topological algorithms were carried out. As shown in Table 2, the top 30 genes of Degree topological algorithm included *DNA topoisomerase II alpha* (*TOP2A*), *THBS1*, and so on. Closer inspection of the table showed *TOP2A* and *proto-oncogene receptor tyrosine kinase Met* (*MET*) are top-ranked in the most topological algorithms. *Maternal embryonic leucine zipper kinase* (*MELK*), *MET*, *THBS1, TOP2A* and *syndecan 1* (*SDC1*) were considered as common hub genes of 12 topological algorithms analysis in the further statistical tests.

### 3.4. Survival Analysis

**Figure 4:**
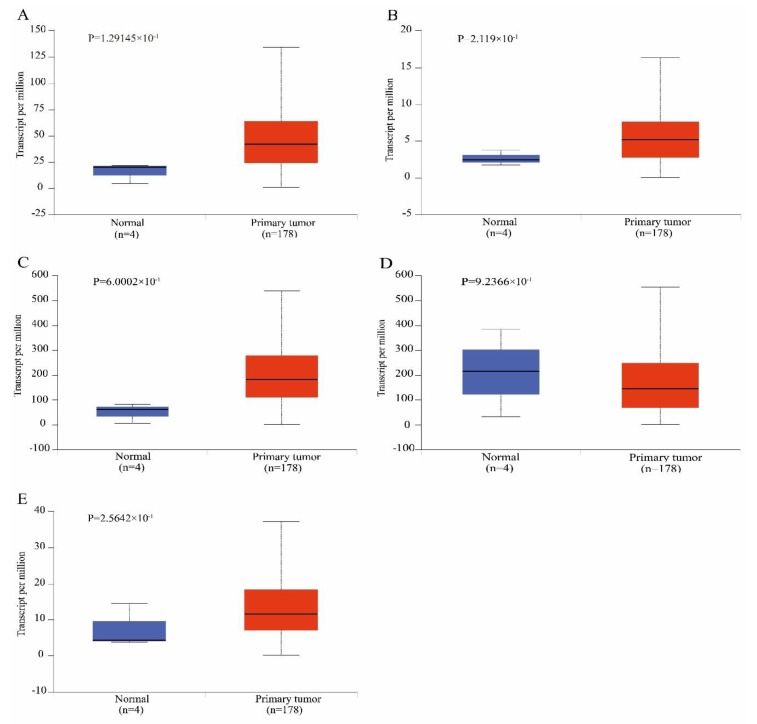
Box-whisker plots showing the expression of hub genes in pancreatic adenocarcinoma samples. (**A**) *Proto-oncogene receptor tyrosine kinase Met*; (**B**) *Maternal embryonic leucine zipper kinase*; (**C**) *syndecan 1*; (**D**) *thrombospondin 1*; (**E**) *DNA topoisomerase II alpha*.

**Figure 5:**
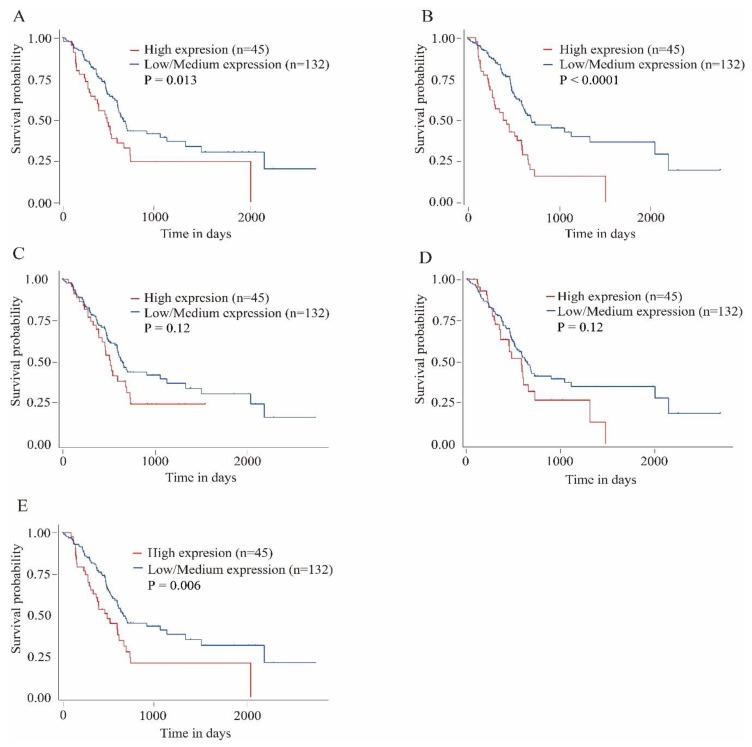
Kaplan–Meier plots showing the association of hub genes expression levels with patient survival. (**A**) *Proto-oncogene receptor tyrosine kinase Met*; (**B**) *Maternal embryonic leucine zipper kinase*; (**C**) *syndecan 1*; (**D**) *thrombospondin 1*; (**E**) *DNA topoisomerase II alpha*.

To validate the reliability of the identified hub genes from the four datasets, UALCAN was used to analyze the hub genes transcript expression and survival in the 182 samples which is derived from the TCGA project. The statistical samples included four normal and 178 pancreatic adenocarcinoma samples. As shown in Figure 4, there was a clear trend of increasing gene expression levels of *MET, MELK, SDC1* and *TOP2A* in primary tumor compared to normal samples. On the contrary, THBS1 was under-expressed in primary tumor. These findings suggested the results of the identified candidate hub genes are reliable. The survival analysis results showed that MELK and TOP2A were linked with poor survival in pancreatic adenocarcinoma (*p* < 0.01). MET was also related with poor survival in the cancer (*p* = 0.013). However, expression levels of SDC1 and THBS1 were not significantly associated with survival probability in the samples, respectively (*p* = 0.12, Figure 5).

## 4. Discussion

During the past decades, many studies have been performed to disclose the causes and underlying mechanisms of pancreatic adenocarcinoma formation and progression. However, the 5-year survival rate for sufferers has only seen slight improvement. Additionally, trustworthy molecular marker with high prognostic value has not yet been determined in pancreatic adenocarcinoma treatment. Due to this disappointing outcome, development of a specific biomarker is urgently needed to detect early pancreatic adenocarcinoma which has a central role in affording patients the best possible outcome. Of equal importance, a new molecular target of drug needs to be identified and validated in order to develop underlying drugs that may be successful in pancreatic adenocarcinoma treatment. Many studies concentrate on an independent genetic event, or the result is generated from independent studies which are inconsistent with each other by microarray analysis [18,19,20]. In this study, the number of up-regulated genes was significantly more than the down-regulated genes (93 vs. 45) for DEG. Previous research reported that the over-expressed genes were markedly more than the under-expressed genes in DEG [21]. Some GEO samples (GSM) of pancreatic adenocarcinoma were not grouped as the cancer group in the cluster analysis. This result may be explained by the fact that these patients were diagnosed as pancreatic cancer patients, and there must be a reason for the lack of clustering such as types of pancreatic adenocarcinoma, different disease subtypes and disease activity or disease stages [22]. Another possible explanation for this is that pancreatic adenocarcinoma pathogenesis in different patients may depend on common changes of the expression of particular critical genes, and rather on personal particular changes of different genes.

There is a growing interest in finding for gene network replace alone genes, contributing to the etiology of complicated diseases, because changes in biological characteristics need interaction in expression of gene sets. The enrichment analysis tool is a beneficial step in this direction for estimating overrepresentation of specific gene category or pathway in a gene list [23]. The results of our analysis showed that DEG was significantly enriched in biological processes including cell migration, cell adhesion, cell-cell adhesion, and extracellular matrix disassembly. One study on gene ontology analysis of the 98 DEG showed that cell adhesion was the main enriched process by genome-scale analysis in patients with pancreatic adenocarcinoma [24]. Moreover, the enriched KEGG pathways of DEG included extracellular matrix receptor interaction and focal adhesion. In human pancreatic adenocarcinoma, both stromal and cancer cells present to be the source of extracellular matrix-degrading metalloproteinase and tissue inhibitor of metalloproteinase [25]. According to KEGG pathway analysis, extracellular matrix (ECM)-receptor interaction was the most enriched pathway in this study. The result was in line with those of a previous study [24]. 

Hub genes, namely *MELK, MET, THBS1, TOP2A* and *SDC1*, were selected with the common parts of 12 topological algorithms in analyzing the DEG PPI network. Notably, only three down-expressed genes, namely *SOCS3, F8* and *THBS1* were displayed in the PPI network. *THBS1* acts as an adhesive glycoprotein that mediates cell-to-cell and cell-to-matrix interaction. In the invasion of human pancreatic adenocarcinomas, *THBS1* was implicated in regulating matrix remodeling and played pivotal role in cancer cell growth and metastasis [26]. Hence, stromal *THBS1* immunoreactivity and expression was considered as a prognostic marker and a new indicator of invasiveness in patients with pancreatic adenocarcinomas [27]. Simultaneously, one observation suggested that metronomic ceramide analogs (C2 and AL6) inhibited angiogenesis in pancreatic cancer through up-regulation of *THBS1* [28]. *THBS1* was significantly decreased in pancreatic cancer patients compared with healthy controls, and low levels of *THBS1* were markedly correlated with poorer survival, preclinically and at clinical diagnosis [29]. Surprisingly, while the expression level of *THBS1* was lower in pancreatic cancer samples than normal, no significant difference was found in the results of this analysis. Survival analysis on individual hub genes disclosed that the survival probability was not obvious between the high expression of *THBS1* and the medium/low expression group. It seems possible that these results are due to multifaceted and sometimes opposing effects of *THBS1* on tumor progression depending on the molecular and cellular composition of the microenvironment [30]. Therapeutic strategy targeting *THBS1* has been widely explored and plentiful peptides and modified structural agents derived from *THBS1* have been developed. For example, compounds of ABT898, and CVX-045 were severally conducted in clinical trials but they were no longer in clinical development due to the adverse events of low objective response rate and slow clearance [31,32]. Trabectedin is a marine natural product. It has been approved for the treatment of advanced or metastatic soft tissue sarcoma and relapsed ovarian cancer. One report indicated that trabectedin displayed anti-angiogenic activity related to the up-regulation of *THBS1* [33]. Trabectedin is currently undergoing phase II clinical trials for several other tumors. 

*SDC1* functions as a transmembrane receptor and engages in cellular proliferation, cell transplantation and cell-matrix interaction. It has previously been observed that *SDC1* expression may play an important role in the pathobiology of pancreatic cancer cell, which is different from that in other gastrointestinal cancers [34]. Notably, while the expression level of *SDC1* was higher in pancreatic cancer samples than normal, survival analysis on separate hub genes revealed that the survival probability was not obvious between the high expression of *SDC1* and the medium/low expression group. These results were likely to be related to the cellular localization of *SDC1* as cell membrane anchored and/or shed, soluble *SDC1* with stromal or nuclear accumulation in individual tumor types [35]. In a human melanoma and ovarian cancer experimental model, the human antibody OC-46F2, specific for the extracellular domain of *SDC1*, blocked vessel maturation and tumor development [36]. The unusual tumorigenic phenotypes resulting from varied *SDC1* expression make it appealing for therapeutic targets. For example, indatuximab ravtansine is a monoclonal antibody-related drug that particularly aims *SDC1*-expressing cells, pre-clinical research corroborated the activity of indatuximab ravtansine in combination with lenalidamide and examethasone for plasma cell myeloma, and a clinical research is continuous [37]. This achievement seems to promote the hopeful results from pre-clinical and clinical researches that studied the chance of therapeutically targeting *SDC1*.

*TOP2A* is one of DNA topoisomerases. Accumulating evidence indicated that it can lead to cancer progression in diverse cancer types, and it has been the certified therapeutic target of anti-cancer and anti-bacterial pharmaceuticals. Recent advances in the field have indicated the feasibility of devising specific-isoform human topoisomerase II poisons, which may be grew as safer anti-cancer pharmaceuticals [38]. In research of forecasting gemcitabine sensitivity with pancreatic adenocarcinomas patient objects, a different expression of *TOP2A* was discovered in gemcitabine sensitive tumors which authenticated as one of potential genes linked with resistance to drug [39]. Survival analysis results of this study showed that *TOP2A* was associated with poor survival in pancreatic adenocarcinoma and the survival probability was obvious between the high expression of *TOP2A* and the medium/low expression group. These results were consistent with another study that discovered that the up-regulation of *TOP2A* was markedly linked with cancer metastasis and smaller survival in adenocarcinomas patient objects [40]. In pancreatic neuroendocrine tumors, *TOP2A* was also identified as one of hub genes by gene microarray analysis [41]. Thereby, *TOP2A* remains as the vital therapeutic target of anti-cancer drugs.

*MELK* was revealed to be commonly up-regulated in differing types of solid tumor, with crucial roles in formation and maintenance of tumor stem cells. In small cell lung cancer and hepatocellular carcinoma, *MELK* expression is consistently elevated in cancer relative to normal tissues [42,43]. This study analysis also exhibited similar results, and *MELK* was linked with poor survival in pancreatic adenocarcinoma. Interesting, survival probability was obviously different between the high expression of *MELK* and the medium/low expression group. Previous results implicated *MELK* can control normal and transformed pancreatic duct cell migration [44]. Although the increase of *MELK* expression has been elucidated in many tumors, no oncogenic variation in the *MELK* gene has been picked out to date. Thus, a small molecule inhibitor of *MELK* that particularly suppresses *MELK* activity may suffer an undesired off-target effect in both normal and tumor cells [45]. Orally administrative *MELK*-targeting compound OTSSP167 inhibited the growth of different types of human cancer including breast, lung, prostate, and pancreas cancer [46]. Hence, inactivation of *MELK* may be therapeutically beneficial.

MET protein is a receptor tyrosine kinase and encoded by *MET* proto-oncogene, also called *tyrosine-protein kinase Met* (*c-Met*). The c-Met kinase has appeared as an appealing target for developing anti-cancer drugs because of its close connection with the generation of diversified human tumors, dismal clinical results and even drug resistance. Active human cancer-linked pancreatic stellate cells caused proliferation and microtube formation of microvascular endothelial cells by *c-MET* signal pathway, which exert a primary effect in human pancreatic adenocarcinoma progression [47]. In stage I-II pancreatic cancer, high *MET* expression was correlated with dismal prognosis and assisted in identifying patients with a high-risk of cancer recurrence and depressing survival prognoses [48]. One report provided evidence that targeting *MET* in combination with gemcitabine may be effective in human pancreatic adenocarcinoma and ensured further clinical evaluation [49]. Blocking the activity of *c-Met* in tumor cells, in combination with other ways for diminishing desmoplasia in the cancer microenvironment, might notably elevate the success of chemotherapy [50]. The mounting evidence demonstrated that *MET* is regarded as a novel therapeutic approach in pancreatic cancer and as a target for personalized therapy [51]. Crizotinib is a tyrosine kinase inhibitor that it can block peritoneal diffusion in pancreatic adenocarcinoma through inhibiting cancer cell proliferation and invasion, at least in part by the suppression of *MET* signal [52]. Hence, it can be speculated that *MET* is a candidate therapeutic target in pancreatic adenocarcinoma and highlighted a collaborative combination of drugs warranting clinical evaluation for pancreatic adenocarcinoma treatment.

## 5. Conclusions

In conclusion, our work has identified 138 DEG in the four profile datasets. DEG significant enriched in biological processes including cell migration, cell adhesion, cell-cell adhesion, extracellular matrix disassembly and several pathways, mainly associated with ECM-receptor interaction, proteoglycans in cancer and focal adhesion pathway in pancreatic cancer. These findings could significantly improve our understanding of the cause and underlying molecular events in pancreatic cancer, these promising molecular markers identified that gene expression profiling studies including *MET, MELK, SDC1, THBS1* and *TOP2A* and pathways could be new effective therapeutic targets for pancreatic cancer.

## Figures and Tables

**Table 1:** Gene ontology and pathway enrichment analysis of differentially expressed genes function in pancreatic cancer (top 5 in each category).

Category	Term	Count	*p*-Value	Genes
BP	GO:0016477 ~ cell migration	10	6.34 × 10^6^	*JUP, STYK1, SDC1, TSPAN1, FAT1, PRKCI, THBS1, SDC4, CEACAM1, ADAM9*
BP	GO:0007155 ~ cell adhesion	13	1.89 × 10^4^	*EFNB2, FERMT1, ITGA2, JUP, LGALS3BP, LAMB3, LAMA3, FAT1, MSLN, LAMC2, THBS1, CEACAM1, ADAM9*
BP	GO:0098609 ~ cell-cell adhesion	9	1.06 × 10^3^	*PKM, FLRT3, S100P, RPL14, CAPG, ANLN, SFN, GPRC5A, CEACAM1*
BP	GO:0022617 ~ extracellular matrix disassembly	5	2.63 × 10^3^	*LAMB3, LAMA3, ADAM10, CAPG, LAMC2*
BP	GO:0031581 ~ hemidesmosome assembly	3	3.56 × 10^3^	*LAMB3, LAMA3, LAMC2*
CC	GO:0070062 ~ extracellular exosome	46	1.35 × 10^7^	*S100A6, TSPO, FXYD3, SLC44A1, TSPAN1, RPL14, MAL2, MARCKSL1, MLPH, SFN, SDC4, GPRC5A, ZG16B, PKM, LGALS3BP, FAT1, CEACAM5, NQO1, THBS1, MYOF, CEACAM1, DLG1, ADAM9, S100P, ADAM10, SLC12A2, LGALS3, TMC5, PRKCI, SLC6A14, S100A10, S100A14, ANXA3, LCN2, JUP, MTMR11, SDC1, KRT19, TNFSF10, LAMA3, C1ORF116, SERPINB5, CAPG, CTSE, SLPI, AOC1*
CC	GO:0005886 ~ plasma membrane	52	3.79 × 10^5^	*SLC44A1, TSPAN1, MARCKSL1, SDC4, PKM, MGLL, CEACAM1, DLG1, NET1, ADAM10, EFNB2, F8, PRKCI, JUP, STYK1, KRT19, SDC1, EFNA5, AOC1, MELK, TNFRSF21, FXYD3, CLDN18, ASAP2, GPRC5A, FAT1, MSLN, AHNAK2, MYOF, FLRT3, KLF5, OSBPL3, SLC12A2, LGALS3, KLB, MET, SLC6A14, ITGA2, DGKH, ITPR3, KCNK1, DOCK5, S100A14, ANXA3, CLDN23, GJB2, KCNN4, P2RX1, PON2, SYTL2, RHBDL2, IFI6*
CC	GO:0005923 ~ bicellular tight junction	7	1.87 × 10^4^	*CLDN18, EPPK1, PRKCI, ECT2, AOC1, CLDN23, DLG1*
CC	GO:0005925 ~ focal adhesion	11	6.35 × 10^4^	*FLRT3, JUP, SDC1, ADAM10, FAT1, EFNB2, FERMT1, FHL2, ITGA2, SDC4, ADAM9*
CC	GO:0005913 ~ cell-cell adherens junction	9	2.70 × 10^3^	*PKM, JUP, S100P, RPL14, CAPG, ANLN, SFN, GPRC5A, DLG1*
MF	GO:0043236 ~ laminin binding	4	7.91 × 10^4^	*LGALS3, ITGA2, THBS1, ADAM9*
MF	GO:0042803 ~ protein homodimerization activity	15	9.42 × 10^4^	*FLRT3, S100A6, ADAM10, S100A10, ECT2, JUP, LCN2, CTSE, MGLL, CEACAM5, AOC1, AGR2, TOP2A, CEACAM1, MTUS2*
MF	GO:0019903 ~ protein phosphatase binding	5	1.18 × 10^3^	*JUP, KCNN4, NEK2, MET, CEACAM1*
MF	GO:0098641 ~ cadherin binding involved in cell-cell adhesion	9	1.35 × 10^3^	*PKM, JUP, S100P, RPL14, CAPG, ANLN, SFN, GPRC5A, DLG1*
MF	GO:0005509 ~ calcium ion binding	13	6.50 × 10^3^	*S100A6, S100P, CAPN8, S100A10, MMP28, ITPR3, S100A14, ANXA3, FAT1, SYTL2, THBS1, AOC1, MELK*
KEGG	hsa04512: ECM-receptor interaction	7	5.33 × 10^3^	*SDC1, LAMB3, LAMA3, ITGA2, LAMC2, THBS1, SDC4*
KEGG	hsa05205: Proteoglycans in cancer	6	1.93 × 10^2^	*SDC1, MET, ITGA2, THBS1, SDC4, ITPR3*
KEGG	hsa04510: Focal adhesion	6	2.17 × 10^2^	*LAMB3, LAMA3, MET, ITGA2, LAMC2, THBS1*
KEGG	hsa00512: Mucin type O-Glycan biosynthesis	3	2.41 × 10^2^	*GALNT5, C1GALT1, ST6GALNAC1*
KEGG	hsa04151: PI3K-Akt signaling pathway	7	5.10 × 10^2^	*LAMB3, LAMA3, MET, ITGA2, EFNA5, LAMC2, THBS1*

Note: BP, biological process; CC, cell component; MF, molecular function; GO, gene ontology; KEGG, Kyoto Encyclopedia of Genes and Genomes (as ranked by the *p*-value).

**Table 2:** The hub genes were analyzed by different topological algorithms in the protein–protein interaction network.

Topological Algorithm	Top 30 Genes Were Ranked by Score
Degree	*TOP2A, THBS1, NEK2, DLG1, ITGA2, MELK, CEP55, ANLN, ECT2, DTL, SDC1, MET, LAMB3, LAMA3, SDC4, PLD1, EFNB2, DGKH, LAMC2, LGALS3, EFNA5, JUP, KRT19, CEACAM5, LGALS3BP, PKM, SGPP2, ADAM10, HS3ST1, MBOAT2*
Edge Percolated Component	*TOP2A, MELK, NEK2, ANLN, DTL, CEP55, ECT2, DLG1, MET, SDC1, THBS1, ITGA2, JUP, SDC4, LAMB3, LAMA3, PKM, EFNA5, LAMC2, KRT19, ADAM10, EFNB2, LGALS3BP, HS3ST1, CEACAM5, PLD1, F8, LGALS3, SLC12A2, PRKCI*
Maximum Neighborhood Component	*TOP2A, NEK2, MELK, CEP55, ANLN, ECT2, DTL, ITGA2, LAMB3, THBS1, SDC1, LAMC2, LAMA3, SDC4, DLG1, PKM, DGKH, EFNA5, MET, JUP, HS3ST1, PLD1, MBOAT2, F8, EFNB2, LGALS3BP, PGM2L1, SDR16C5, PRKCI, FHL2*
Density of Maximum Neighborhood Component	*CEP55, ANLN, ECT2, DTL, NEK2, MELK, LAMC2, LAMA3, TOP2A, LAMB3, ITGA2, SDC4, DLG1, PKM, DGKH, EFNA5, MET, JUP, HS3ST1, PLD1, MBOAT2, F8, EFNB2, LGALS3BP, THBS1, SDC1, PGM2L1, SDR16C5, PRKCI, FHL2*
Clustering Coefficient	*PKM, CEP55, ANLN, LAMC2, ECT2, DTL, HS3ST1, MBOAT2, F8, NEK2, MELK, LAMB3, LAMA3, DGKH, EFNA5, SDC4, JUP, LGALS3BP, SDC1, ITGA2, TOP2A, THBS1, PLD1, EFNB2, MET, DLG1, PGM2L1, SDR16C5, PRKCI, FHL2*
Maximal Clique Centrality	*TOP2A, NEK2, MELK, CEP55, ANLN, ECT2, DTL, ITGA2, THBS1, DLG1, TNFSF10, LAMB3, LAMA3, SDC1, OAS1, IFI6, IFI27, LAMC2, MET, SDC4, PLD1, EFNB2, DGKH, LGALS3, JUP, KRT19, CEACAM5, EFNA5, LGALS3BP, GALNT5*
Bottleneck	*MET, DLG1, TOP2A, SDC1, KRT19, PLD1, MELK, THBS1, LGALS3, ITGA2, EFNB2, DGKH, SGPP2, SDC4, JUP, S100A6, CEACAM5, LCN2, PKM, CEP55, ANLN, PGM2L1, SDR16C5, PRKCI, LAMB3, LAMC2, ECT2, LAMA3, DTL, NEK2*
EcCentricity	*MET, DLG1, PRKCI, SDC1, TOP2A, EFNA5, ADAM10, JUP, STYK1, KRT19, EFNB2, MELK, PKM, CEP55, ANLN, PGM2L1, SDR16C5, ECT2, THBS1, DTL, NEK2, LGALS3, SLC12A2, RHBDL2, SDC4, FAT1, HS3ST1, PLD1, FOXQ1, ITGA2*
Closeness	*TOP2A, DLG1, MET, MELK, SDC1, THBS1, ITGA2, NEK2, JUP, CEP55, ANLN, ECT2, DTL, PLD1, KRT19, EFNB2, SDC4, EFNA5, CEACAM5, ADAM10, PKM, LAMB3, LGALS3, PGM2L1, SDR16C5, SLC12A2, PRKCI, STYK1, LGALS3BP, LAMA3*
Radiality	*MET, DLG1, TOP2A, SDC1, MELK, KRT19, JUP, EFNB2, EFNA5, THBS1, ADAM10, ITGA2, PRKCI, STYK1, PLD1, CEACAM5, SDC4, NEK2, CEP55, ANLN, ECT2, DTL, LGALS3, PKM, PGM2L1, SDR16C5, SLC12A2, HS3ST1, FAT1, LAMB3*
Stress	*MET, DLG1, TOP2A, SDC1, THBS1, PLD1, KRT19, ITGA2, LGALS3, MELK, EFNB2, CEACAM5, S100A6, LCN2, SDC4, LGALS3BP, JUP, DGKH, SGPP2, ADAM10, EFNA5, LAMB3, LAMA3, NEK2, PKM, CEP55, ANLN, PGM2L1, SDR16C5, PRKCI*
Betweenness	*MET, DLG1, TOP2A, SDC1, PLD1, THBS1, KRT19, ITGA2, LGALS3, EFNB2, MELK, CEACAM5, DGKH, SGPP2, JUP, S100A6, LCN2, LGALS3BP, SDC4, EFNA5, ADAM10, LAMB3, LAMA3, NEK2, PKM, CEP55, ANLN, PGM2L1, SDR16C5, PRKCI*
Common genes of 12 topological algorithms	*MET, MELK, SDC1, THBS1, TOP2A*

## Supplementary Materials

The following are available online at https://www.mdpi.com/2073-4425/10/8/612/s1, Figure S1: hierarchical clustering with limiting the analysis only to the 138 common DEGs. The row denotes the genes, the column denotes the samples, **A**: GSE15471, **B**: GSE19650, **C**: GSE32676, **D**: GSE71989, Table S1: Analysis gene ontology and KEGG pathway by KOBAS 3.0; Table S2: Definition of 12 topological algorithms.
