# Supplementary material for: Identification of Key Genes and Pathways in Pancreatic Cancer Gene Expression Profile by Integrative Analysis
Source: Genes (Basel). 2019 Aug 13;10(8):612. doi: 10.3390/genes10080612 (PMC6722756; doi:10.3390/genes10080612)
Supplement: Supplementary file 1 [file genes-10-00612-s001.pdf]

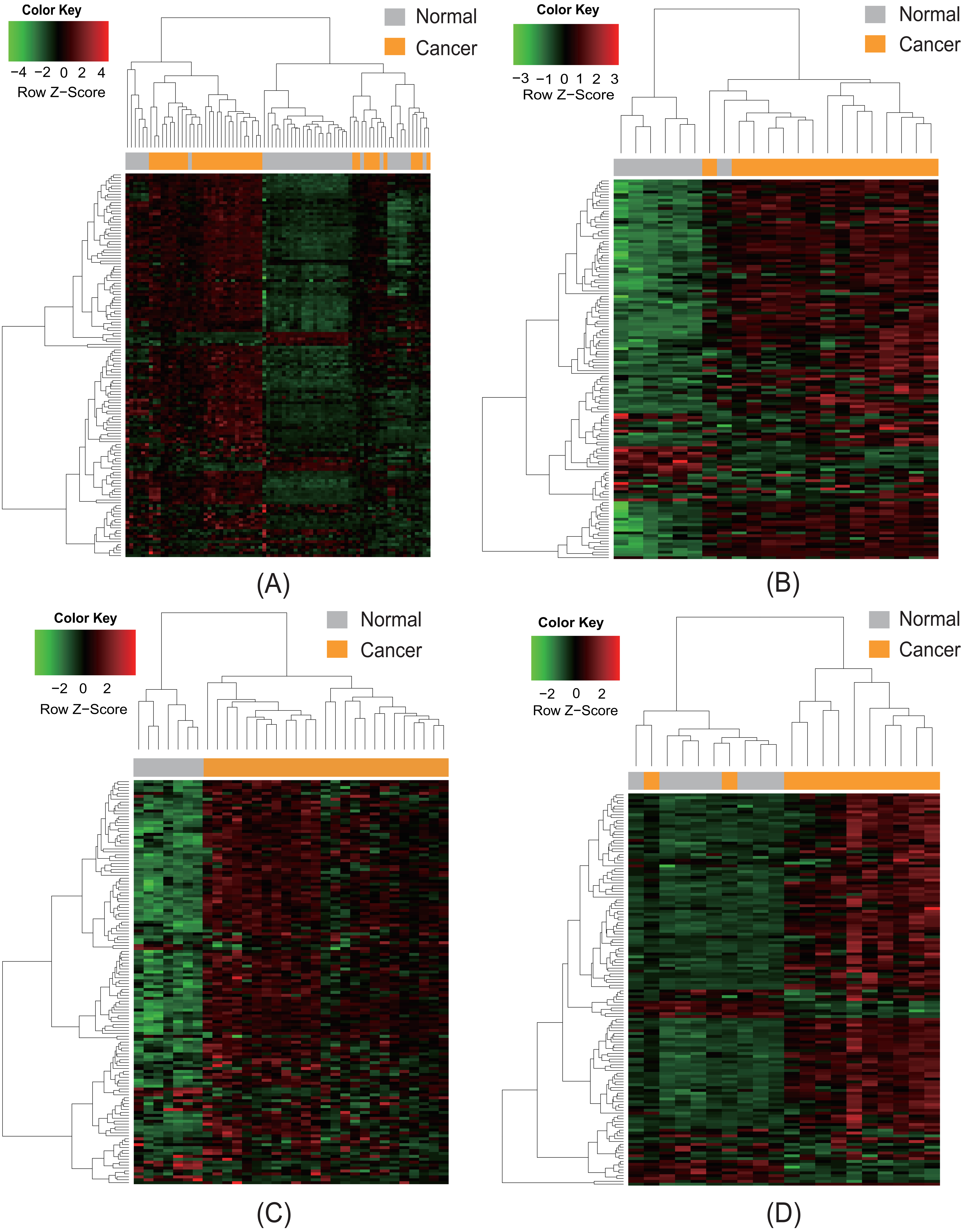
Color Key
−4 −2 0 2 4
Row Z−Score
Normal
Cancer
(A)
Color Key
−3 −1 0 1 2 3
Row Z−Score
Normal
Cancer
(B)
Color Key
−2 0 2
Row Z−Score
Normal
Cancer
(C)
Color Key
−2 0 2
Row Z−Score
Normal
Cancer
(D)

Supplementary table 1. Definition of 12 topological algorithms

Algorithm	explanation
Clustering Coefficient	The global clustering coefficient is based on triplets of nodes. A triplet is three nodes that are connected by either two (open triplet) or three (closed triplet) undirected ties. A triangle graph therefore includes three closed triplets, one centered on each of the nodes (n.b. this means the three triplets in a triangle come from overlapping selections of nodes). The global clustering coefficient is the number of closed triplets (or 3 x triangles) over the total number of triplets (both open and closed).
We assume that a biological network $G = (V, E)$ is an undirected network, where V is the collection of nodes within the network and E is the edge set. We can use another notation $G = (V(G), E(G))$ to represent a network, where $V(G)$ is the collection of nodes in a network G, and $E(G)$ is the collection of edges in a network G. For a set S, we use $\|S\|$ to denote its cardinality (i.e. the number of elements in the set). Local based method only considers the direct neighborhood of a vertex. Given a node v, $N(v)$ denotes the collections of its neighbors.	
Degree method (Deg)	$Deg(v) = \|N(v)\|$
Maximum Neighborhood Component (MNC)	$MNC(v) = \|V(MC(v)\|$<br>where $MC(v)$ is a maximum connected component of the $G[N(v)]$ and $G[N(v)]$ is the induced subgraph of G by $N(v)$.
Density of Maximum Neighborhood Component (DMNC)	$DMNC(v) = \|E(MC(v))\|/\|V(MC(v))\|^{\varepsilon}$<br>where ε = 1.7.
Maximal Clique Centrality (MCC)	Given a node v, the MCC of v is defined as $MCC(v) = \sum_{C \in S(v)} (\|C\| - 1)!$, where $S(v)$ is the collection of maximal cliques which contain v, and $(\|C\| - 1)!$ is the product of all positive integers less than $\|C\|$. If there is no edge between the neighbors of the node v, then $MCC(v)$ is equal to its degree.
The length of a shortest path between nodes u and v is denoted as $dist(u, v)$. Let $C(v)$ be the component which contains node v.	
Closeness (Clo)	$$Clo(v) = \sum_{w \in V} \frac{1}{dist(v, w)}$$
EcCentricity (EC)	$$EC(v) = \frac{\|V(C(v))\|}{\|V\|} \times \frac{1}{max\{dist(v, w): w \in C(v)\}}$$
Radiality (Rad)	$$Rad(v) = \frac{\|V(C(v))\|}{\|V\|} \times \frac{\sum_{w \in C(v)} (\Delta_{C(v)} + 1 - dist(v, w))}{max\{dist(v, w): w \in C(v)\}}$$<br>where $\Delta_{C(v)}$ is the maximum distance between any two vertices of the component $C(v)$.
BottleNeck (BN)	Let T_s be a shortest path tree rooted at node s. $BN(v) = \sum_{s \in V} p_s(v)$ where $p_s(v) = 1$ if more than $\|V(T_s)\|/4$ paths from node s to other nodes in T_s meet at the vertex v; otherwise $p_s(v) = 0$.
Stress (Str)	$Sre(v) = \sum_{s \neq t \neq v \in C(v)} \sigma_{st}(v)$, where $\sigma_{st}(v)$ is the number of shortest paths from node s to node t which use the node v.

Betweenness (BC)	$BC(v) = \sum_{s \neq t \neq v \in C(v)} \frac{\sigma_{st}(v)}{\sigma_{st}}$, where σ_{st} is the number of shortest paths from node s to node t.
Edge Percolated Component (EPC)	Given a threshold (0 ≤ the threshold≤ 1), we create 1000 reduced networks by assigning a random number between 0 and 1 to every edge and remove edges if their associated random numbers are less than the threshold. Let the G_K be the reduced network generated at the *k*th time reduced process. If nodes u and v are connected in G_k, set δ_{vt}^{k} to be 1; otherwise $\delta_{vt}^{k} = 0$. For a node v in G, $EPC(v)$ is defined as $EPC(v) = \frac{1}{\lvert V \rvert} \sum_{k=1}^{1000} \sum_{t \epsilon V} \delta_{vt}^{k}$.

Supplementary table 2. Analysis gene ontology and KEGG pathway by KOBAS 3.0

Term	Database	ID	Gene number	Gene entrzed ID	P-Value
ECM-receptor interaction (ranked 1)	KEGG PATHWAY	hsa04512	7	3914|7057|3909|6385|6382|3673|3918	2.76×10^{-8}
Focal adhesion (ranked 2)	KEGG PATHWAY	hsa04510	6	3914|3909|7057|3673|3918|4233	9.30×10^{-5}
Proteoglycans in cancer (ranked 3)	KEGG PATHWAY	hsa05205	6	3710|7057|6385|6382|3673|4233	9.80×10^{-5}
Mucin type O-Glycan biosynthesis (ranked 4)	KEGG PATHWAY	hsa00512	3	11227|56913|55808	2.24×10^{-4}
PI3K-Akt signaling pathway (ranked 5)	KEGG PATHWAY	hsa04151	7	3914|3909|1946|7057|3918|3673|4233	2.24×10^{-4}
cell migration (ranked 60)	Gene Ontology	GO:0016477	29	10276|706|57402|55612|3909|6385|2195|5337|6382|3673|10103|5584|6286|79148|7057|7262|23767|54443|80005|3958|634|8754|55359|1948|306|3728|102|4233|6374	1.42×10^{-16}
cell adhesion (ranked 91)	Gene Ontology	GO:0007155	26	10276|137075|55612|3909|7057|6385|10232|3958|2195|10551|3673|1739|1948|1946|6281|23767|51208|3959|3914|634|8754|27242|3918|3728|102|1048	4.97×10^{-12}
cell-cell adhesion (ranked 264)	Gene Ontology	GO:0098609	13	1739|1948|1048|634|8754|137075|1946|27242|23767|3958|3728|2195|51208	1.33×10^{-5}
extracellular matrix disassembly (ranked 429)	Gene Ontology	GO:0022617	4	102|3914|3909|3918	2.90×10^{-4}
hemidesmosome assembly (ranked 275)	Gene Ontology	GO:0031581	3	3914|3909|3918	1.79×10^{-5}
extracellular exosome (ranked 53)	Gene Ontology	GO:0070062	46	6590|114569|706|57402|8743|79098|7057|6385|79838|2195|6382|9052|23446|10103|1739|5584|6286|6558|6281|5315|124220|65108|3959|3958|634|2810|8754|3934|79083|26|306|3909|26509|3728|102|9045|3880|1728|6277|11254|1048|5349|5268|10903|1510|822	7.67×10^{-20}
plasma membrane (ranked 45)	Gene Ontology	GO:0005886	64	25924|114569|54843|3710|3775|10276|1946|57402|8743|6281|6385|10232|3958|2195|5337|11343|3673|9052|23446|10103|1739|5584|152831|54933|9833|6286|6558|7057|5315|23767|2157|51208|9462|113146|65108|27242|6382|5023|137075|634|8754|55612|55359|2706|26|306|26509|3728|160851|102|4233|3783|3880|6277|11254|1048|5349|5445|26031|55450|688|8853|2537|1948	6.19×10^{-23}

bicellular tight junction (ranked 227)	Gene Ontology	GO:0005923	6	1739|5584|137075|26|51208|1894	3.50×10^{-6}
focal adhesion (ranked 169)	Gene Ontology	GO:0005925	11	2274|1948|8754|55612|6385|23767|3728|2195|6382|102|3673	1.61×10^{-7}
cell-cell adherens junction (ranked 2592)	Gene Ontology	GO:0005913	1	3728	1.76×10^{-1}
laminin binding (ranked 239)	Gene Ontology	GO:0043236	4	8754|3673|3958|7057	5.86×10^{-6}
protein homodimerization activity (ranked 178)	Gene Ontology	GO:0042803	14	26|1048|634|6277|23767|11343|7153|6281|1894|3728|10551|102|23281|1510	2.72×10^{-7}
protein phosphatase binding (ranked 343)	Gene Ontology	GO:0019903	5	634|3783|4233|3728|4751	7.74×10^{-5}
calcium ion binding (ranked 203)	Gene Ontology	GO:0005509	13	26|54843|3710|6277|388743|9833|6286|57402|79148|7057|306|6281|2195	1.09×10^{-6}

Note: The term of gene ontology is ranked in brackets by KOBAS when they are ranked in top 15 by DAVID analysis. The term of KEGG pathway is ranked in brackets by KOBAS when they are ranked in top 5 by DAVID analysis.
